# Exploring barriers and facilitators to water availability and accessibility, and potential strategies for improving water accessibility and children’s intake in family childcare homes: a qualitative study

**DOI:** 10.3389/fnut.2025.1442070

**Published:** 2025-03-12

**Authors:** Kim M. Gans, Violeta Chacón, Sarah Wen Warykas, Madeline Baird, Vanessa Esquivel, Suge Zhang, Alison Tovar, Snehaa Ray, Naomi Inman, Peter McCauley, Viviana C. Zambrano Rodriguez, Michelle Miller, Nathaniel Stekler, Patricia Markham Risica

**Affiliations:** ^1^Department of Human Development and Family Sciences, University of Connecticut, Storrs, CT, United States; ^2^The UConn Rudd Center for Food Policy and Health, Hartford, CT, United States; ^3^Department of Anthropology, University of Connecticut, Storrs, CT, United States; ^4^Department of Behavioral and Social Sciences, Center for Health Promotion and Health Equity, Brown School of Public Health, Providence, RI, United States; ^5^Department of Nutritional Sciences, University of Connecticut, Storrs, CT, United States; ^6^School of Journalism and Mass Communications, College of Information and Communications, University of South Carolina, Columbia, SC, United States

**Keywords:** children, childcare, water, hydration, families, feeding, beverages

## Abstract

**Background:**

Children in the U.S. drink too little water and too much juice and sugar sweetened beverages. Inadequate access to drinking water in locations where children spend substantial time, like family childcare homes (FCCH) could play a role in low child water intake. The aim of this qualitative study was to explore barriers and facilitators to water availability and accessibility in FCCH, and determine potential strategies for facilitating water accessibility and children’s intake in FCCH.

**Methods:**

We conducted virtual interviews, in Spanish and/or English, with family child care providers (FCCP) from Rhode Island, Connecticut, and Massachusetts. Interviews were conducted by University of Connecticut graduate students, including two who were fluent in Spanish and English. These were audio-recorded, transcribed verbatim, and translated to English. We conducted a deductive analysis using *a priori* themes. Additional codes were developed and applied to capture emerging themes from the qualitative data.

**Results:**

Twenty FCCP (100% identified as female; 50% as Latina) participated in the interviews. FCCP barriers to water availability at FCCH included focus on other beverages, e.g., milk; confusion with the Child and Adult Care Food Program (CACFP) guidelines regarding water, and concerns about: water quality, mess, children eating enough food/milk, bathroom accidents, and cost for filters/bottled water. Barriers to children drinking water included: children not liking or preferring water, parental preferences/role modeling, and parental concerns about water quality. Suggested potential strategies to facilitate water access and intake included water filters to ensure safe water access, self-serving stations and water bottles to encourage autonomy among children, and incorporating water into daily routines. Participants also favored materials and activities to educate and encourage children to drink water and to keep track of their intake.

**Conclusion:**

These findings suggest that interventions to increase water consumption at FCCH should provide resources to guarantee safe water access to children, encourage children to drink water, and help clarify misperceptions and confusion around CACFP beverage guidelines. Future research should evaluate the effectiveness of interventions to provide education and water access resources to FCCP and families on improving child water access, availability and intake.

## Introduction

Adequate water consumption supports children’s overall health and cognitive function (1–5). Water is also a healthy, low-cost beverage alternative to sugar-sweetened beverages (SSB). Habitual water drinkers eat a better quality diet (including a lower intake of SSB) than non-water drinkers (6–8). In addition, adequate water consumption, instead of SSB, is associated with lower calorie, sugar, sodium, and saturated fat consumption (9, 10), which can in turn help prevent health conditions such as obesity, and diet-related chronic diseases like Type-II diabetes, stroke, and heart disease (11–15).

While drinking water is critical for all age groups, it is particularly important for young children as they are more vulnerable to dehydration (16–18). Unfortunately, children in the U.S. drink too little water and too much juice and SSB (19, 20). According to national data from 2005 to 2010, 28% of children aged 4–13 did not drink plain water on two consecutive days (21). Moreover, one in two children in the U.S. is inadequately hydrated and one in six children does not drink any tap water (22, 23). Low water consumption and high SBB intake is more pronounced among low-income and racial/ethnic minority populations compared with White populations (10, 23, 24).

Furthermore, children develop dietary preferences at an early age (25); thus, exposing them to water, rather than SSB and juice, may help promote a preference for water (26–28). This is important because SSB intake is associated with higher body mass index (29, 30) and higher adipose tissue in children (31). Small shifts in caloric intake can help prevent obesity, for example, by eliminating SSB in the diet and replacing with plain water. Consuming water instead of SSB can also reduce the risk of dental caries in young children (32–34). Furthermore, a recent review found a connection between inadequate daily water intake with kidney stones and type 2 diabetes in adults (35), which further suggests the importance of consuming enough water throughout life.

Inadequate access to drinking water in locations where children spend substantial time could play a role in low water intake (22, 36). Childcare settings are an important health promotion venue for young children with 75% of U.S. children in childcare for an average of 35 h/week (37). While some research has been done regarding water access and availability in childcare (38–46) very little has been done in family childcare homes (FCCH), which care for close to 2 million US children (37, 47–50). Families from low-income and ethnic minority backgrounds may prefer FCCH over other childcare settings due to cultural preferences for family-like care, flexible hours and lower costs (51–53). As a result many family childcare providers (FCCP) care for children from low-income, ethnic minority families, and often share similar socioeconomic and ethnic backgrounds (48–50). Young children who spend time in FCCH may be at higher risk for obesity (54, 55), which highlights the need to further study these settings.

A recent study surveyed childcare providers including FCCP and found that drinking water was not commonly served or offered to children at meals or snacks (56). While reasons for this are unclear, data from our own observations in a previous nutrition and physical activity intervention trial with 120 FCCH in Rhode Island and Massachusetts (72% Latino) found that at baseline (in 2015–2018) only 17% of FCCP made drinking water available at all times and only 7% prompted children to drink water during indoor/outdoor playtime (38) Most (94%) of FCCH had drinking water available from the faucet tap or refrigerator dispenser; but only 14% had drinking water in the room with the children (i.e., in a pitcher with cups); only 9% had drinking water outside and only 30% had child self-service water available (38). In a separate pilot study, we also collected observational data in 20 FCCH caring for infants and toddlers less than age 2 and found even lower access to water (39). Thus, there appears to be a need for improvement in water availability and access at FCCH, but the reasons for this low water access and how to improve it are unknown. Perceived barriers should be explored with FCCP along with potential strategies to improve water accessibility and children’s water intake in FCCH (36).

The purpose of this study was to conduct qualitative interviews with FCCP to explore barriers and facilitators to water availability and accessibility in FCCH, and determine potential strategies for facilitating water accessibility and children’s intake in FCCH. This research will shed light on the drivers related to beverage availability in FCCH and will inform future environmental and policy approaches to improving the childcare water/beverage environment as well as young children’s water intake.

## Materials and methods

The work completed for this formative research study is Phase 1 of a two phase project funded by Robert Wood Johnson Foundation Healthy Eating Research (Grant #283-4135). Phase 1 consisted of qualitative interviews with ethnically diverse FCCP in Connecticut, Rhode Island and Massachusetts to understand their attitudes, barriers and practices regarding water/beverage access and availability in their FCCH, and potential strategies. These findings informed Phase 2, an intervention study (Drink Well/Bebe Bien) to pilot environmental strategies to improve water availability and accessibility in FCCH (to be reported later). The study was reviewed and approved by the institutional review board at the University of Connecticut (Storrs, CT).

FCCP were recruited for the qualitative interviews using the following methods. We contacted community organizations/networks that provide training and support to FCCP and provided recruitment flyers/emails for these organizations to distribute to their FCCP. We also sent direct mailings (Connecticut) or emails (Rhode Island and Massachusetts) to licensed FCCP in Rhode Island, Connecticut, and Massachusetts state databases. We also called some FCCP in these databases. Word of mouth referrals also came from FCCP already participating in the study.

Interested FCCP were provided with a QR code on the flyer or link via email to connect to a brief screening survey on Qualtrics to see if they were eligible to participate in the interview. To be eligible, providers needed to operate a FCCH in Rhode Island, Connecticut or Massachusetts, care for at least 2 children ages 6 months–5 years old, speak English or Spanish, and be at least 18 years old. We focused on preschool age children (age 5 and under) because they are more likely than school age children to spend multiple hours and mealtimes in childcare. We also started at age 6 months because infants usually should not drink plain water until they are at least 6 months old (57).

If participants were eligible, the Qualtrics survey took them to an information sheet/consent form with details of the study. Once participants read the consent form and provided consent to participate, they were directed to complete another survey, which collected demographic information about the provider (gender, age, ethnicity, educational level, years working as a FCCP), and information about the FCCH (how many children they care for; whether the FCCH is enrolled in CACFP, how many hours children are cared for per week, and the state that the FCCH operates in). The survey also asked about the best days and times for an interview. Research assistants contacted participating FCCP via email or phone to schedule the interview. Then, a Webex link was sent to the participants along with a reminder of the date and time. Participants were also sent reminders the day before the interview via email, text message, and/or phone call—depending on the participant’s preference.

The interviews were conducted from April 2022 to October 2022 by University of Connecticut graduate students and were audio-taped. Interviews were conducted in Spanish or English based on participant preference by the bilingual research team. At the end of the interview, participants received an electronic code for a $40 Amazon gift card. Then, participants were asked to complete a recontact form via a link in the Webex chat to indicate whether or not they were interested in being recontacted for future research opportunities.

### Interview guide

Investigators (KG, AT, PR) and graduate students (VZR, VE, SW) developed the interview guide. The moderator guide focused on 6 domains (Table 1), which were driven by the aims of this study to identify barriers, challenges and facilitators/strategies to providing water to children in FCCH and potential strategies to improve water availability, accessibility, and children’s intake, which would be used to inform the Phase 2 intervention study.

**Table 1:** Interview script domains, questions, themes, and quotes from family childcare provider interviews.

Domain and questions	Themes	Representing quotes
General beverage perceptions (milk, juice, water, SSB) What are your thoughts about what beverages children in childcare should be drinking?	Beverages served in childcare based on nutrition (CACFP) program guidelines	*“I always let parents know about the nutrition program I am a part of. It’s where I receive education to serve children the healthiest food possible at the daycare. So, I tell them that they always have milk at breakfast, snacks are a bit more flexible, but I always offer milk with lunch and dinner. I always offer them water. In the cases where I offer them juice, I make sure it’s 100% juice that has been approved by the nutrition program.”*
		*“I always give them milk for lunch or breakfast. I also give them water with breakfast or lunch, and many times I do offer them water when they are having a snack. Juice can be once or twice a week, but I offered them milk every day either four or three times a day. The same goes with water, I offer it to them three or four times a day.”*
	*Milk most important beverage*	*“Sometime a lot of the kids at lunch, they get about a half a cup of milk and they do not usually drink all of it… So sometimes for afternoon snack, if they did not drink much of their milk for lunch. I offer it again at snack and the kids that obviously finished their milk, they will then get water.”*
		*“Well my kids get milk with breakfast and lunch. And water with their snacks”*
	Some FCCP serve no juice to children, others limit juice in childcare or serve as a treat	*“So, we are very limited in the amount of 100% pure juice that we give them. I do not remember if it’s just twice a week that we can give them 100% juice. But I try to not give them any juice at daycare, I am not very familiar with juice. I mostly give them water and milk, especially with this heat.”* “I’ve had occasional children ask me for juice, or something, you know. I just simply tell the kids we do not drink that at [Childcare name]…this is what we drink at [Childcare Name].” “I definitely do not think juice. I know what they should not be drinking is juices. Other than that, for what I believe is what we do, is either milk or water.”
		*“Juice is a very big treat in our house.”*
		*“Sometimes parents will throw a juice box for a special treat for them.”*
		*“Once in a while, we have some kind of a little holiday party, and they get watered down apple juice.”*
Barriers to serving water in childcare	Water quality/safety – many providers had concerns about water quality; others did not	*“They [childcare providers] do not know if the water is clean if they do not have a filter. These are some concerns that keep providers from offering water.”* *“The water quality may not be the best with certain homes, like the town water or the city water versus well water.”* *“Well, if I lived in Michigan, I would definitely be concerned after all that trouble they had with their water… But basically, if the water smells funny, then I would have a concern. So, I really do not – my water does not smell funny and does not taste funny. So, I do not have a concern personally. But if I did, then I would contact my water department.”*
What concerns might providers have about serving water as a beverage with a meal or providing water throughout the day for children when they are thirsty? Tell me about any challenges you might have faced with children having access to drinking water in your home	Taste as a barrier	“*So, our water – it tastes like chlorine if we do not have the filter, and no one likes it. Even my children, they will not drink water from the faucet. Even brushing their teeth, they – they do not like doing it. Sometimes we do bottled water, but that’s mainly to go. But they normally just use their cups. If you use water to make ice cubes, you could taste the chemicals in it too. It’s too much of a chemical taste compared to filtered water or bottled water.”*
	Cost as a barrier	*One barrier is a big financial if you want to offer clean water and you want to have these access stations, you – it requires filters, it requires buying the actual little stations. So… it was about $60, which is still a lot and could be a lot for childcare providers…”*
	Bathroom accidents may occur with child water consumption	*“I do not announce, “Who wants a water break?” because again, it’s a distraction. Because they all want to do it. Then it’s also – because I use five- ounce cups, you have eight different children, that’s a water break every hour, that’s a lot of cups we are going through.”*
		*“…some childcare providers might be concerned with children having to go to the bathroom more often if they are drinking a lot of water, depending on the age. If one child has to go to the bathroom, then everyone has to go inside, or something like that.* *That type of worry, children going to the bathroom more often…”*
	*Concerns about Mess, Lack of Space Safety*	*“Accidents during nap time. If they are drinking a lot in the morning. They typically nap after lunch so that the water intake on top of the milk could cause accidents.”* *“Probably the biggest barrier would be the fact that it is our homes. So, they are not allowed to bring it into my living room, which we do use for a short portion of the day. And, in the playroom and such, they have, like I said, some kind of sippy cup, straw cup, something so that it’s not an empty cup to just knock over.”*
		*“Some providers get upset with spills if the kids are learning to drink from open cups…”*
		*“I do not know that a lot of homes have room for, like, a water station or go help yourself because I think a lot of kids are, it’s, it’s going to be not just a mess, but could kind of be a safety issue.”*
	*Parents not always supportive of water*	*“He does not have a water bottle in his backpack and he always complains…. I talked to her about getting a water bottle, but she did not.”*
	*Child Won’t Eat if Drink Water*	*“I’ve had an occasion where parents said, can I send in a juice box for lunch? I allow it because… if that’s what the parent wants to send as part of their lunch. It’s not me serving it. I look at, you know, the parents choosing. I Only had so much control over the children”*
	*Divergence about CACFP water guidelines. Some FCCP confused*	*“If they are drinking their whole cup of water before lunch, and then they do not eat their lunch.* *“Well, the beverages, like the water, I just continuously give them access to it. I do not really have an amount that they are supposed to be drinking. With the milk, it’s through the food program. They have specific guidelines for the child’s age”.*
		*“We have a food program through CACFP, and even if they do not want to participate, our regulations state that they need to offer healthy, nutritious meals and snacks. I’m not 100% sure that water is written in there…”* *“I think there are specific amounts – I do not know if it’s for water. I know it’s for type of milk goes by age through our regulations through the state. Depending on the age, they say so many ounces. Or if they are younger, then a certain age they have to have whole milk. But for water, I do not think there’s a certain regulation or anything that we really have to follow specifically.”* *“Aside from that, as home daycare providers, as a part of the food program, they do a training for you. They have information on their website. They have videos, and you have to always read the updates….The food program specifies, for example, you have to give them a glass of milk in the morning or with lunch. You have to offer them at least one glass of milk a day. Or it says every snack should be accompanied by water. So I think a lot of it is that you learn from the rules from the state and the food program to be able to give it to the children. “*
Solutions to serving water in childcare	Importance of building water breaks into routine	*“To me, the most important part is to incorporate it [water] into a routine and keep water available at all times…especially with the heat during summer.”*
What are some ideas for helping providers serve more water to children during meals and snacks? What might help providers remember to prompt children to drink water during indoor and outdoor playtime?		*“I think in order for these kids to have more of an appreciation for water, we need to make it part of our routine, part of our transitions”* *“Our day is pretty chopped up anyway. We do a lot with transition. We’re very busy with a lot of different activities. We could include a water break during our transitions so that’s not really interrupting a lot”.* *“Yes, so you make it [water] a part of their routine…” “That way, it’s part of their routine and it’s something you do every day.”*
		*“Well, the way I do it is I structure it so we actually have official water breaks.”*
	Providing self-serve water access for child autonomy	*“In my opinion, the most important part is to keep a consistent routine. For example, you could have a station prepared for the children, one that is appropriate for their ages. I do not have one right now but it sounds like a very good idea.”*
		*“That self-serving station is a good idea because our job is to make children independent. And they love to do things by themselves…”* *“…maybe if we go outside, then have one of the self- serve water containers, where they could push the little spout and then it comes out in their cup; teach them that.”* *“I have all my clients provide some kind of water bottle. And they have water throughout the whole day, because their water bottle is full, and it will sit on my counter…If we are outside, we bring the water bottles outside. And during our courses of play, every 15 min, I make them stop and take a water break…”*
Barriers to child water consumption in childcare About a third of children in the United States do not drink any plain water over a 2 day period. What are some reasons why you think so many children do not drink water?	Parental influences on child water intake: lack of water access at home, parents do not role model water drinking; parents serve sugary drinks and juice’ parents lacking knowledge of healthy beverages	*“Because it is not given to them. More than likely. It’s not given to them. Parents just give them all that sugary stuff.”* *“Their parents do not drink it [water]. The parents do not model for them.”* “*A lot of kids are not brought up from a very young age, drinking water.”* *“There are so many people that automatically serve juice.”* *“Parents also not being aware of the importance of drinking water and the impact of sugary drinks”*
		*“Many times, if a parent like myself does not drink any water then the children are going to follow their example and not like water either. That’s why it’s very important to remind them to drink water because it’s important to them.”*
		*“It’s a whole struggle to give them [children] water in the afternoon because some parents give them apple juice…”*
		*“Sometimes parents give them [children] juice because they want to make them happy. They get used to that and they do not drink water…”* *“But kids listen, and they listen when parents are talking to teachers. They know what’s going on. So, if a parent says to the teacher, ‘Well, Johnny does not drink water, so if you could put milk in his cup for the day, that would be great,’ then Johnny’s never gonna drink water because mom said Johnny does not drink water and he heard it”.*
	Taste of water	*“Well, because it [water] has no taste. I’ve also heard adults do not drink water for this exact reason. They need to add flavoring to water in order to drink it. So, I think that if children do not drink water, it’s*
	*Water quality/safety*	*because they say they do not like it because it has no taste…”* *“To have the child say, I do not like water.” “It does not have a lot of flavor.”* *“But, if kids have never been really offered it, and they are always being given, you know, milk and/or juice or whatever, water does not taste good when you are used to some of that, too.”* *“Well, they do not like the water. I mean, it does not have a taste most of the kids like something that tastes good.”* *“Uh, parents not wanting the kids to drink water or unsure if tap water was clean and safe”.* *“So, our water – it tastes like chlorine if we do not have* *the filter, and no one likes it. Even my children, they will not drink water from the faucet.”* *Yeah, I think with the daycare children, you could tell that they definitely drink less water if it’s tap water. There’s been times where I’m in the middle of filtering out the – the what’s it called – the filter. And you have to filter it so many times before you can actually use it and I’ve given them sink water and they do not want to touch it. They’ll take a sip and they are like “Ew,” and they’ll put it down, and they’ll just leave it. The only one who gets the sink water is my dog…”*
Solutions to improving child water consumption	Role modeling water drinking and educating children and parents on water benefits	*“…the biggest part is just making it known the importance of water and just continue. Even though someone does not like it, you just gotta keep encouraging, encouraging, and modeling is the big thing and modeling having the parents model at home. Everyone has to model because that’s how you are gonna get children to really drink more water.* *“I think that if the children do not drink too much water, then it’s on us as adults to promote those habits. We have to give them the options. Because many times…children are so used to drinking juice that it’s very hard to make a transition from juice to water. So, an example, if you do not want to see Pedrito cry, he might be given juice behind the nutrition program’s back. So, then how are you going to teach the child to drink water? So, in those cases, I talk to the parents about the importance of cutting back on the amount of juice. And I ask them to start mixing it with water. That way the child can get used to not having the strong taste of juice. Because the moms buy these juices that are full of sugars and artificial colors”.* *So, as providers, we have to be consistent and tell them that water is healthier and that it’s what it’s allowed in daycare instead of juice. And if one day we do it, but then do not follow through the next day, then the child is not going to learn because I have not had that issue. I am not going to say that it’s not hard when the children are new here, but it’s a transition that is not long.* *Parents, they do not have that awareness about – it was never – they are not aware of how important it is for not only them to drink water but their children. Some parents may not, or people in general just may not have that knowledge. Like, “Oh. I did not know water was so important. I just drink juice, coffee, but water. I never knew it was…” So, it’s like they are not aware. They need to be educated or some type of awareness about the importance of consuming water.* *“Yeah, cause it could be a generational thing where it wasn’t important in their home. Because I have not always thought water was important to drink. Until I, yeah, became a provider and learned about nutrition and health. So, it would be nice if parents or people, in general, could have access to this information to bring about an awareness, because what’s common and normal and natural for some is just like totally oblivious to some people also. That make sense?”* *“I think we [childcare providers] need to explain to children that water is important and that they need to drink water throughout the day so they are hydrated.”*
What are some ideas for getting children to drink more water in childcare?		*“We’re asking you to teach them [children] about their body. Teach them about healthy choices. This the education part of it is helpful too.”* *“Well… purchase some educational books.”*
	Visual reminders for water consumption	*“Well, I personally have some cups that when you add ice to them, the colors turn brighter. So, they are encouraged by this to drink more water. When they see that the cup turns a different color, because it’s cold, I think it helps them drink more water.”*
		*”Yeah, so visual reminders. So, I know even some providers have mentioned a poster somewhere that has a water design…”*
		*“…supplying them with fun cups, whether they are character cups or kid-friendly cups. I think they would be excited about that. Maybe certain little markers on their cups…”*
		*“I had to use visual aids to help children understand that they have to drink water. Because water is a resource for the body.”*
Needs/wants for intervention Our plan is to develop a program/intervention that could include a free package of equipment, materials and services that we would deliver to family childcare homes to help them serve more water to children and get them to drink it. What should go into this package?	Water-related materials to use with children like reward charts, books, coloring pages, stickers, posters	*“Some kind of tracking chart of how many glasses of water your child drinks at home. And the child could put a star or a water droplet sticker next to it, and then bring it back to the provider so we know if they are drinking water at home. Because if they are going to drink water at home, they are going to drink water here.”*
	Kid-friendly drinking cups/bottles; kid- sized pitchers	*“Books about water.”* *“Beverage posters or beverage activities”* *“Kids love stickers, if we could find stickers…maybe like a raindrop that shows water…;* *“Coloring books…there’s different coloring pages.”* *“Some nice drinking cups that have the straw that closes. Or the little, smaller kid-sized pitchers that they can fill with water and put on the table for the kids to learn self-sufficient…”*	*“Maybe having little bottles that are kind of cute.” “Small kids-sized bottles to facilitate them to drink more.”*	*“Supplying the programs with a case of little water bottles.”*	Water storage resources and water filters	*“Yeah, water filters, they definitely help. I use one that’s specific to my refrigerator, but maybe if it’s a Brita or something that’s more specific for daycare and that actually goes towards the pitcher. So, it’s a pitcher and water filter combined…”*	*Water filters are expensive”. I have to pay for them myself.*	*A cooler to take water outside, or depending on your situation, even in your house…”*	*“Access to water storage materials like cups, fill up water bottles, self-serving stations, water filters.”*

### Analysis

Interview recordings were transcribed verbatim by a professional transcription service and those conducted in Spanish were translated to English. A data code book was developed based on study objectives to document barriers and solutions to improve water access and consumption in FCCH. Dedoose software (version 9.0.107) was used to code qualitative data for further analysis. Based on the interview guide and study objectives, transcripts were coded using structural coding to categorize interview data. Using the structural codes, the transcripts were coded and reviewed by the research team to carry out iterative phases of data analysis.

Concepts and themes were then reviewed in various phases by the research team to ensure that all of the *a priori* and emergent themes were captured. Based on this review, additional codes were developed and applied to capture emerging themes from qualitative data. These themes were then discussed with the entire research team and finalized. See Table 1 for interview script domains, interview questions, themes, and sample quotes from FCCP interviews. A subset of sample quotes are also provided in the text in the Results section. Descriptive statistics were computed from the survey data, using SPSS software (version 29; IBM Corp, Armonk, NY). We examined responses across states but did not find substantive differences in responses to the interview questions from providers in different states; therefore, themes and quotes are not separated by state.

## Results

A total of 20 FCCP participated in the interviews. While we initially planned to interview up to 36 FCCP, recruitment and scheduling challenges extended the timeline. However, data saturation was reached with 20 participants, and therefore, no additional interviews were conducted. All participants identified as female; 50% identified as Hispanic (Table 2). The majority of participants (70%) were from Connecticut, with five participants (25%) from Rhode Island and 1 participant (5%) from Massachusetts. Mean age was 49 years, and 65% had at least some college education. On average, providers cared for 7 children and had cared for children in their homes for an average of 17 years. A total of 70% of FCCP reported participating in CACFP.

**Table 2:** Sociodemographic characteristics of interview participants (*n* = 20).

	*n* (%)
Female	20 (100)
Age, mean ± SD years	49 ± (12.88)
Hispanic/Latinx	10 (50)
Education	
High school	7 (35)
Some college	7 (35)
College or more	6 (30)
Number of children in FCCH, mean ± SD	7 ± (1.97)
Number of years working in the childcare profession, mean ± SD	17 ± (11.40)
Number of hours per week that children spent in their childcare, mean ± SD	44 ± (8.35)
Participating in CACFP	14 (70%)
State of residence	
Connecticut	14(70)
Rhode Island	5(25)
Massachusetts	1(5)

FCCH, family childcare homes; CACFP, Child and Adult Care Food Program.

The duration of interviews ranged from 26 to 53 min (mean 40.9 min, median 42 min). Qualitative results are presented below according to the interview guide domains. Themes are incorporated within each of the domains with supporting quotes, as appropriate (also see Table 1).

### Providers’ perceptions on beverages served in childcare

FCCP were asked about their perceptions on what beverages children in childcare should be drinking. Providers who were part of the federal CACFP reported following specific dietary guidelines regarding beverage options for children. Provider perceptions on milk, water and juice offerings in childcare varied depending on child beverage preferences and meal-time routines. All providers mentioned serving milk often and many stressed the importance of serving milk to children. Some providers talked about serving different types of milk to children of different ages. Water was often mentioned as a beverage offered after milk or during snacks, although some FCCP offered water throughout the day.

*“I always let parents know about the nutrition program I am a part of. It’s where I receive education to serve children the healthiest food possible at the daycare. So, I tell them that they always have milk at breakfast, snacks are a bit more flexible, but I always offer milk with lunch and dinner. I always offer them water. In the cases where I offer them juice, I make sure it’s 100% juice that has been approved by the nutrition program.”*

*“…at lunch, they get about a half a cup of milk and they don't usually drink all of it… So sometimes for afternoon snack, if they didn't drink much of their milk for lunch. I offer it again at snack and the kids that obviously finished their milk, they will then get water.”*

*“Well, my kids get milk with breakfast and lunch. And water with their snacks.” “Well, in the case of milk, it depends on the child’s age. It has to be low-fat. It shouldn’t contain any fat. Now, if the child is younger than 2 years of age, then you don’t have to give him milk with [low] fat content. He needs to drink whole milk. And if they are babies, then you give them formula…”*

Although there was consensus on milk (and secondarily water) as the most crucial beverage for children to consume in FCCH, there was some divergence among participants regarding juice. Some providers believed serving juice was appropriate if served in limited amounts, during specific periods such as snack time, for special occasions, or if diluted with water. On the contrary, other providers were adamant about not serving juice in their FCCH. For example, one FCCP shared, *“I’ve had occasional children ask me for juice, or something, you know. I just simply tell the kids we don’t drink that at [Childcare name]…this is what we drink at [Childcare Name]”. Similarly, another provider stated that she limits beverage options to only water or milk, stating, “I definitely don’t think juice. I know what they shouldn’t be drinking is juices. Other than that, for what I believe is what we do, is either milk or water”.*

Other FCCP talked about juice being served occasionally and as a treat, *“Once in a while, we have some kind of holiday party, and they [children] get watered down apple juice.”. Other participants shared similar sentiments, “Sometimes, parents will throw a juice box for a special treat for them [children]”; or “Juice is a very big treat in our house.”*

### Providers perceptions on barriers to serving water in childcare

We asked providers what concerns they had about serving water to children and any challenges they faced with children having access to drinking water in the FCCH. Safety/cleanliness of water in FCCH was an area where there was divergence. Multiple providers expressed concern about water safety/cleanliness in the childcare, and emphasized the need for water filters. For example, one provider stated: *“They [childcare providers] do not know if the water is clean if they do not have a filter. These are some concerns that keep providers from offering water.” Another provider stated: “The water quality may not be the best with certain homes, like the town water or the city water versus well water.”* However, other FCCP were comfortable that the water in their FCCH was safe: For example, one FCCP stated:

*“Well, if I lived in Michigan, I would definitely be concerned after all that trouble they had with their water… But basically, if the water smells funny, then I would have a concern. So, I really don’t – my water doesn’t smell funny and doesn’t taste funny. So, I don’t have a concern personally. But if I did, then I would contact my water department.”*

Another barrier was the taste of the water, which could be improved by using filters. As one FCCP stated:

*“So, our water – it tastes like chlorine if we don’t have the filter, and no one likes it. Even my children, they will not drink water from the faucet. Even brushing their teeth, they – they don’t like doing it. Sometimes we do bottled water, but that’s mainly to go. But they normally just use their cups. If you use water to make ice cubes, you could taste the chemicals in it too. It’s too much of a chemical taste compared to filtered water or bottled water.”*

While filters could help with water safety and taste, they also added cost, which was mentioned as another barrier:

*“One barrier is a big financial if you want to offer clean water and you want to have these access stations, you – it requires filters, it requires buying the actual little stations. So, which in our case, it was about $60, which is still a lot and could be a lot for childcare providers…”*

Some childcare providers were concerned that an increase in water access may be associated with an uptick in bathroom frequency and/or accidents.

*“Some childcare providers might be concerned with children having to go to the bathroom more often if they’re drinking a lot of water, depending on the age. If one child has to go to the bathroom, then everyone has to go inside, or something like that. That type of worry, children going to the bathroom more often.”*

Many childcare providers also expressed concerns about children serving themselves water as it could cause water spills and mess. It was also mentioned that water spills could increase safety concerns within the FCCH.

*“Probably the biggest barrier would be the fact that it is our homes. So, they’re not allowed to bring it into my living room, which we do use for a short portion of the day. And, in the playroom and such, they have, like I said, some kind of sippy cup, straw cup, something so that it’s not an empty cup to just knock over.”*

*“Some providers get upset with spills if the kids are learning to drink from open cups…”*

*“I don't know that a lot of homes have room for, like, a water station or go help yourself because I think a lot of kids are, it's, it's going to be not just a mess, but could kind of be a safety issue.”*

Some FCCP were concerned that unlimited water access might impact children’s appetite and displace food consumption.

*“If they're drinking their whole cup of water before lunch, and then they don't eat their lunch.”*

Some FCCP mentioned that parents were not always supportive of their children drinking water, and preferred juice, or that parents did not support water availability in childcare by bringing water bottles in for their children.

*“He [the child] doesn't have a water bottle in his backpack and he always complains…. I talked to her [his mom] about getting a water bottle, but she didn't.”*

*“I've had an occasion where parents said, can I send in a juice box for lunch? I allow it because… if that's what the parent wants to send as part of their lunch. It's not me serving it. I look at, you know, the parent’s choosing. I only had so much control over the children.”*

There was divergence in understanding the CACFP guidelines regarding serving children water. Some FCCP shared that they knew that CACFP had guidelines about milk, but they were unsure what was recommended regarding water. One provider stated, “*We have a food program through CACFP, and even if they do not want to participate, our regulations state that they need to offer healthy, nutritious meals and snacks. I’m not 100% sure that water is written in there…*.” Another provider shared, *“I think there are specific amounts – I do not know if it’s for water. I know it’s for type of milk goes by age through our regulations through the state. Depending on the age, they say so many ounces. Or if they are younger, then a certain age they have to have whole milk. But for water, I do not think there’s a certain regulation or anything that we really have to follow specifically.”* Other FCCP were more clear about the CACFP guidelines for water, so this wasn’t a barrier for them. One FCCP stated:

*“Aside from that, as home daycare providers, as a part of the food program, they do a training for you. They have information on their website. They have videos, and you have to always read the updates….The food program specifies, for example, you have to give them a glass of milk in the morning or with lunch. You have to offer them at least one glass of milk a day. Or it says every snack should be accompanied by water. So I think a lot of it is that you learn from the rules from the state and the food program to be able to give it to the children. “*

### Providers’ perceptions on solutions to serving water to children in childcare homes

When we asked FCCP about their ideas for helping providers serve more water to children and what might help them remember to prompt children to drink water during playtime, multiple FCCP emphasized the importance of incorporating water into a structured routine and water breaks throughout the day.

*“I think in order for these kids to have more of an appreciation for water, we need to make it part of our routine, part of our transitions”.*

*“Our day is pretty chopped up anyway. We do a lot with transition. We’re very busy with a lot of different activities. We could include a water break during our transitions so that’s not really interrupting a lot”.*

Some childcare providers stressed the importance of child autonomy on water access and intake. Using self-serving stations or individual child water bottles were suggestions that could allow children to readily access water on an unlimited basis.

*“That self-serving station is a good idea because our job is to make children independent. And they love to do things by themselves.”*

*“I have all my clients provide some kind of water bottle. And they have water throughout the whole day, because their water bottle is full, and it will sit on my counter…If we’re outside, we bring the water bottles outside. And during our courses of play, every 15 minutes, I make them stop and take a water break…”.”*

### Provider perceptions on barriers to child water consumption in childcare homes

FCCP felt that parents played a significant role in determining whether children consumed water. They highlighted the availability of water outside the childcare setting, particularly at children’s homes. Some FCCP expressed concerns that water was not readily accessible at home and noted that some parents provided SSB and juice as alternatives to water.

*“Because it is not given to them. More than likely, it’s not given to them. Parents just give them all that sugary stuff.”*

*“Sometimes parents give them [children] juice because they want to make them happy. They get used to that and they don’t drink water…”*

FCCP also emphasized the importance of positive parental role modeling on child eating and drinking behaviors. They reported that parents often model drinking unhealthy beverages like sugary drinks and juice and do not role model drinking water with their children.

*“Many times, if a parent like myself doesn’t drink any water then the children are going to follow their example and not like water either. That’s why it’s very important to remind them to drink water because it’s important to them.”*

*“But kids listen, and they listen when parents are talking to teachers. They know what’s going on. So, if a parent says to the teacher, ‘Well, Johnny doesn’t drink water, so if you could put milk in his cup for the day, that would be great,’ then Johnny’s never gonna drink water because mom said Johnny doesn’t drink water and he heard it”.*

FCCP also emphasized how water quality and safety/cleanliness impacted beverage choices for children at childcare and at home. They also mentioned that water quality affected the taste of the water and that children were deterred from drinking water due to its lack of taste. Plain water was refused by some children who preferred other drinks over water.

*“Uh, parents not wanting the kids to drink water or unsure if tap water was clean and safe”.*

*“Yeah, I think with the daycare children, you could tell that they definitely drink less water if it’s tap water. There’s been times where I’m in the middle of filtering out the – the what’s it called – the filter. And you have to filter it so many times before you can actually use it and I’ve given them sink water and they don’t want to touch it. They’ll take a sip and they’re like “Ew,” and they’ll put it down, and they’ll just leave it. The only one who gets the sink water is my dog…”*

*“Well, because it [water] has no taste. I’ve also heard adults don’t drink water for this exact reason. They need to add flavoring to water in order to drink it. So, I think that if children don’t drink water, it’s because they say they don’t like it because it has no taste…”*

### Provider perceptions on solutions to improving child water consumption

When asked about strategies to encourage children to drink more water in childcare, FCCP emphasized the importance of educating children on the benefits of water consumption for their health. They also suggested using child-friendly materials and visual cues/reminders to promote water intake. Specific ideas included providing personalized and character-based cups, brightly colored and appealing kid-friendly water bottles, and reward charts to encourage water intake within FCCH.

*“I think [childcare providers] need to explain to children that water is important and that they need to drink water throughout the day so they are hydrated.”*

*“We’re asking you to teach them [children] about their body. Teach them about healthy choices. This the education part of it is helpful too.”*

*“…supplying them with fun cups, whether they’re character cups or kid-friendly cups. I think they would be excited about that. Maybe certain little markers on their cups…”*

*“Yeah, so visual reminders. So, I know even some providers have mentioned a poster somewhere that has a water design…”*

Similarly, providers mentioned the importance of having adults (FCCP and parents) role model drinking water and FCCP providing education for both children and parents on the importance of water.

*“…the biggest part is just making it known the importance of water and just continue. Even though someone doesn't like it, you just gotta keep encouraging, encouraging, and modeling is the big thing…having the parents model at home. Everyone has to model because that's how you're gonna get children to really drink more water”.*

*“I think that if the children don’t drink too much water, then it’s on us as adults to promote those habits. We have to give them the options. Because many times…children are so used to drinking juice that it’s very hard to make a transition from juice to water… So, in those cases, I talk to the parents about the importance of cutting back on the amount of juice. And I ask them to start mixing it with water. That way the child can get used to not having the strong taste of juice. Because the moms buy these juices that are full of sugars and artificial colors”.*

*“Yeah, cause it could be a generational thing where it wasn’t important in their home. Because I haven’t always thought water was important to drink. Until I, yeah, became a provider and learned about nutrition and health. So, it would be nice if parents or people, in general, could have access to this information to bring about an awareness…””*

### Provider preferences for intervention resources

We informed FCCP about our plan to develop an intervention that would include a free package of equipment and materials to help them serve more water to children and encourage water consumption. We then asked FCCP for their input on what should be included in the intervention package. FCCP expressed interest in water-related materials to use with children such as child-friendly tracking/reward charts including stickers to help track child water intake throughout the day. FCCP also mentioned posters, books, coloring pages and water-related activities to do with the children.

*“Some kind of tracking chart of how many glasses of water your child drinks at home. And the child could put a star or a water droplet sticker next to it, and then bring it back the provider so we know if they’re drinking water at home. Because if they’re going to drink water at home, they are going to drink water here.”*

*“Beverage posters or beverage activities.”*

Providers mentioned that having child-friendly drinking cups/bottles and pitchers would help them to promote water intake and allow children to access water more easily themselves.

*“Some nice drinking cups that have the straw that closes. Or the little, smaller kid- sized pitchers that they can fill with water and put on the table for the kids to learn self-sufficient…”*

*“Maybe having little bottles that are kind of cute.” “Small kids-sized bottles to facilitate them to drink more.”*

Supplying FCCH with water filters was also deemed helpful to help decrease water contamination and ensure water quality and safety, although filter cost could be a barrier. FCCP also mentioned water storage resources like self-serving water stations or coolers.

*“Yeah, water filters, they definitely help. I use one that’s specific to my refrigerator, but maybe if it’s a Brita or something that’s more specific for daycare and that actually goes towards the pitcher. So, it’s a pitcher and water filter combined…”*

*“Water filters are expensive.” I have to pay for them myself.”*

*“Access to water storage materials like cups, fill up water bottles, self-serving stations, water filters.”*

## Discussion

The purpose of this qualitative study was to explore the barriers to water availability/accessibility and children’s water intake in FCCH and determine potential strategies to facilitate water accessibility and intake. One important finding in this study is the confusion around CACFP beverage guidelines among some FCCP, which has been reported in previous studies with both FCCP and childcare centers (44, 45). CACFP requires participating childcare sites to make potable water available to children upon request throughout the day and, also, to offer water to children throughout the day (43). According to the CACFP meal pattern, providers are not permitted to serve water as a substitute for milk during meals. However, water can be offered alongside milk at mealtimes and is recommended as a beverage option during snacks when milk is not served (43). However, some FCCP in our study were unclear about the CACFP water guidelines. This is consistent with evidence that shows that some childcare providers have a misperception that CACFP does not allow for water to be served alongside milk during meals (40). This may discourage them from serving water at meals and snacks at their FCCH. Several studies in California have shown that CACFP childcare providers were less likely to provide water compared to non-CACFP providers (40, 42), which may also indicate confusion with CACFP guidelines regarding water. As the states of RI, CT and MA all require licensed childcare providers to follow CACFP nutrition guidelines whether or not providers participate in CACFP, understanding CACFP beverage guidelines is important for all providers.

To encourage more water consumption among children at FCCH, the USDA should consider providing clearer written communication about the CACFP beverage guidelines to reassure FCCP that water is not intended to compete with milk. In addition, the USDA, state departments of education, CACFP monitors/fiscal sponsors, and/or FCCH network organizations could offer training sessions to reinforce the beverage guidelines and provide practical strategies for making water accessible to children throughout the day, alongside the recommended foods and milk in the CACFP guidelines (43). State licensing could also incorporate stronger policy language regarding water availability, complementing existing policies on other beverages. For example, in California, state policies mandating access to drinking water throughout the day in childcare settings were associated with increases in childcare facilities offering water with meals and snacks, as well as making water easily and visibly available to children to self-serve (44, 45). These policy changes were accompanied by awareness campaigns underscoring the importance of not only implementing policies but also ensuring that childcare providers fully understand them.

In addition to misperceptions of CACFP guidelines, some FCCP expressed their concern about the safety of tap water served at FCCH and parents’ perceptions that tap water is not safe to drink. This finding is consistent with other studies in the U.S. reporting consumer perceptions that tap water might not be safe to drink, with such perceptions more likely among people of color, including Black and Hispanic parents (36, 58, 59). Water safety is a multifactorial issue including quality of water from public water systems, presence of lead in tap water, and poor water organoleptics (e.g., taste, appearance, temperature) that are interpreted as unsafe water or that make water unappealing to drink, and may cause misperceptions of tap water safety. All of these issues were relevant to the FCCP in our study. In some cases, concerns about water safety are warranted given documentation of lead and/or other contaminants in US drinking water, with water quality violations more common in low-income communities of color than in higher income White, non- Hispanic communities (36). However, there may also be unwarranted fear of poor water quality/safety causing financial burden to FCCH and families for purchasing bottled water or water filters as most public water systems in the US are safe (36).

The three New England states that participated in this study have routine testing for microbiological, chemical, and toxicological contaminants in public water systems (60–62). The Safe Drinking Water Act, which specifies the amounts of contaminants or disinfectants allowed in drinking water from public water systems, including those in childcare settings, is enforced by the Environmental Protection Agency (EPA) (63). These standards apply to public water systems that supply tap water in most FCCH. FCCH with private water sources like a well are required to test water and report problems to the state (64). According to the EPA’s Drinking Water Performance and Results Report, 94% of Rhode Island’s, 94% of Connecticut’s, and 97% of Massachusetts’ public drinking water systems were in compliance with the maximum contaminant levels (65).

Furthermore, Rhode Island, Connecticut and Massachusetts all require water safety testing of childcare facilities and offer technical assistance and resources to childcare facilities for testing and follow-up measures related to lead and other contaminants in drinking water (61, 66–69). The most common sources of lead in drinking water are lead pipes, faucets, and fixtures as well as lead services lines connecting the home to the water main (70). Lead can enter drinking water when plumbing materials that contain lead corrode, especially where the water has high acidity or low mineral content (70). This is particularly a problem in older homes and buildings, which may be more of an issue in low-income areas (71). In 2020, the U.S. EPA announced new funding to test for lead in schools and childcare facilities located in low-income and disadvantaged communities authorized under the Water Infrastructure Improvements for the Nation (WIIN) Act (72), which could help FCCH improve water quality. More recently, the Infrastructure Investment and Jobs Act of 2022 (also known as the Bipartisan Infrastructure Law) appropriated additional funds for testing and remediation of lead in school and childcare drinking water (73). Furthermore, the new Lead and Copper Rule Improvements (released by the EPA on 10/30/24) specify actions to inventory, remove and replace lead service lines (74).

Recommended policies and practices to address both real and perceived issues related to water safety and quality include coordinated efforts at the municipal, state and federal levels to improve water safety and quality. These efforts should include monitoring tap water safety, implementing remediation measures in childcare facilities when necessary, and providing training and communication to ensure that FCCP are aware of and utilize available resources. Additionally, public campaigns should be developed and promoted to effectively communicate information about water safety and to address and correct misperceptions about water quality (36). Future research should include assessing tap water contamination in childcare facilities and evaluating the cost and effectiveness of remediation strategies (36), identifying optimal messaging strategies to communicate about water safety and change public misperceptions when water is safe to drink especially among lower-income communities and populations of color (36).

In addition to confusion about CACFP guidelines and water quality, other barriers that were identified in the current study have been found in other studies surveying both FCCH and childcare centers. These include lack of CACFP reimbursement for water (44, 45), cost of supplies (44, 45), concerns about reduced milk or food intake (44), children’s taste preferences for less healthy beverages (45) and parents bringing sugar-sweetened beverages to child care sites (45). Barriers that were not mentioned in these prior studies but were identified in the current study included concerns about mess, lack of space, and safety when children self-access water, and bathroom accidents occurring with increased child water consumption. Such barriers might be specific to FCCH given their unique environments in home settings with a single provider caring for multiple children of different ages.

Participating FCCP in our study mentioned several potential strategies to facilitate water access and intake in FCCH that might overcome these barriers such as incorporating water into the daily feeding and activity routines at FCCH and having child-appropriate equipment, like water bottles or cups with straws that close, and spill-proof kid-size pitchers at self-serve water stations that would support children’s autonomy but decrease concerns about mess, safety and space. Research has shown that equipment and visual materials can help caregivers to create supportive environments for healthy feeding practices, including water intake (75, 76). For example, a cross-sectional survey among childcare centers in several U.S. states found that child-size pitchers and cups support the feeding environment (76). Autonomy-supportive feeding practices, which offer a structured environment where children can participate in making their own food choices such as ensuring water is always available and encouraging children to drink water during playtime, are associated with better diet quality among children at FCCH (77, 78). Furthermore, findings from two school-based interventions suggest that increasing water consumption can reduce obesity-related costs and promote public health (79, 80).

FCCP in our study also mentioned that water-themed educational materials/activities for kids would be helpful. Few child care–based interventions have focused on increasing drinking water intake among children. During a summer campaign in California, the Bay Area Physical Activity and Nutrition Collaborative distributed coloring pages, activity worksheets, and books about drinking more water to child care facilities (81, 82). Although the effect of this campaign on childcare participants was not directly examined, an evaluation of the campaign’s impact on the overall population demonstrated that nearly 50% of individuals who received educational materials reported that they were drinking fewer SSB and sports drinks after exposure to the campaign (81). More water-related intervention studies are needed for childcare settings, especially for FCCH. Based on the results of the current study, such interventions should include water resources for the FCCH such as water filters, self-serve water stations, and child water bottles in addition to training and educational resources for FCCP.

Interventions that seek to increase water intake among children in childcare should also consider parents and the child’s home environment. FCCP specifically mentioned the need to encourage water promotion and consumption within the home. Interventions that provide water filters to families have been shown to reduce parents’ concerns about water insecurity and even improve their organoleptic perceptions of water (83, 84). A 2020 systematic review and meta-analysis on the effectiveness of lifestyle interventions to increase children’s water consumption found that on average, children in intervention groups consumed 29 mL/d more water than did children in control groups (44). This effect was larger in interventions explicitly focused on diet (73 mL/d), than in interventions focused also on other lifestyle factors (44). Since then, a more recent intervention providing water filters to Latinx families along with education significantly reduced children’s SSB and increased water consumption (75, 84). More studies are needed to evaluate the effectiveness of interventions that provide education and water access resources to improve child water access, availability, and intake both in childcare and at home.

This study has some limitations that are worth mentioning. FCCP were invited to participate in the study through a Qualtrics link and interviews were conducted virtually using Webex. Therefore, we might not have reached FCCP without internet access or those who do not have the technological proficiency to use these channels. The study was conducted in three New England states and only included FCCP who spoke English or Spanish, so the results may not be generalizable to other states or FCCP speaking other languages. The study data is all qualitative from interviews, which are subjective and can be vulnerable to bias. In future studies it would be helpful to combine such self-reported qualitative data with objective observations of water access as well as measurement of children’s water intake in FCCH.

In conclusion, this study provides evidence of the challenges and barriers that FCCH face in providing water to children, which may differ from larger centers. In addition to the perceptions of FCCP, future studies could also include the viewpoints of parents and the state agencies that supervise or license FCCH to be able to triangulate the results and get a more complete picture of how to improve water access and water consumption of young children.

## Data Availability

The raw data supporting the conclusions of this article will be made available by the authors, without undue reservation.
